# G-Quadruplex in Gene Encoding Large Subunit of Plant RNA Polymerase II: A Billion-Year-Old Story

**DOI:** 10.3390/ijms22147381

**Published:** 2021-07-09

**Authors:** Adriana Volná, Martin Bartas, Václav Karlický, Jakub Nezval, Kristýna Kundrátová, Petr Pečinka, Vladimír Špunda, Jiří Červeň

**Affiliations:** 1 Department of Physics, Faculty of Science, University of Ostrava, 710 00 Ostrava, Czech Republic; adriana.volna@osu.cz (A.V.); vaclav.karlicky@osu.cz (V.K.); jakub.nezval@osu.cz (J.N.); 2 Department of Biology and Ecology, Institute of Environmental Technologies, Faculty of Science, University of Ostrava, 710 00 Ostrava, Czech Republic; martin.bartas@osu.cz (M.B.); P18116@student.osu.cz (K.K.); petr.pecinka@osu.cz (P.P.); 3 Global Change Research Institute, Czech Academy of Sciences, Bělidla 4a, 603 00 Brno, Czech Republic

**Keywords:** evolution, plant science, nucleic acids, circular dichroism, UV light

## Abstract

G-quadruplexes have long been perceived as rare and physiologically unimportant nucleic acid structures. However, several studies have revealed their importance in molecular processes, suggesting their possible role in replication and gene expression regulation. Pathways involving G-quadruplexes are intensively studied, especially in the context of human diseases, while their involvement in gene expression regulation in plants remains largely unexplored. Here, we conducted a bioinformatic study and performed a complex circular dichroism measurement to identify a stable G-quadruplex in the gene *RPB1*, coding for the RNA polymerase II large subunit. We found that this G-quadruplex-forming locus is highly evolutionarily conserved amongst plants sensu lato (Archaeplastida) that share a common ancestor more than one billion years old. Finally, we discussed a new hypothesis regarding G-quadruplexes interacting with UV light in plants to potentially form an additional layer of the regulatory network.

## 1. Introduction

G-quadruplexes (G4s) in nucleic acids are noncanonical four-stranded structures, which are different from classical double-stranded DNA (B-DNA form) described in 1953 by James Watson, Francis Crick, and Rosalind Franklin [1,2]. The basic building block for a G4 is a so-called guanine quartet formed by a G:G Hoogsteen base pairing, a structure which was first proposed by Gellert and his colleagues in 1962 [3,4]. G4 formation usually requires monovalent cations with a positive charge, such as potassium (K^+^) and sodium (Na^+^) ions [5]. It has been demonstrated that G4s have the potential to frequently occur in specific genomic loci, generally called G4-forming sequences or putative G4 sites. These regions are widely found in various eukaryotes [6,7,8], prokaryotes [9,10,11], and even viruses [12,13,14,15]. There is direct evidence of the functional relevance of such a structure; that is, G4s generally slow the replication process and induce instability during leading-strand replication [16,17], affect transcription by arresting RNA polymerase [18,19,20], and stop translation of the protein if a stable G4 is formed in the transcribed mRNA [19,21]. There are also studies suggesting that G4s are sensitive to UV light in vitro [22,23]. However, G4s are still a neglected area in plant research when compared to humans and model animals. Limited knowledge on the topic was reviewed in [24] stating the unknown function of most plant G4s. In this study, we inspected the *RPB1* gene, which encodes the large subunit of RNA polymerase II. RNA polymerase II (DNA directed RNA polymerase II) is usually associated with transcription of most structural genes. Eukaryotic RNA polymerase II consists of 12 subunits encoded by different genes [25,26], and in 2005, an exact 3D structure of RNA-polymerase II from *Saccharomyces cerevisiae* was resolved [27]. The activity of RNA polymerase II is precisely regulated on several levels, but there are large differences among species, especially between mammals and plants. For example, mammalian RNA polymerase II is localized to the so-called transcription factories in the nucleus, while in plants, RNA polymerase II is rather more evenly distributed in the nucleoplasm [28]. Moreover, in plants, specialized polymerases have evolved (RNA polymerases IV and V, and organelle-specific polymerases), so the specificity of substrates slightly differs [29]. Recently, a novel role of plant RNA polymerase II has been described—they silence retrotransposons and, thus, maintain genome stability [30]. RNA polymerase II is a multi-subdomain complex; the number of domains, as well as their positions, differs between plant species, which points to a complicated evolution for this enzyme, as reviewed in [26]. RNA polymerase protein complexes are considered to be one of the main regulators of gene expression processes in all living organisms [31,32]. Thus, we decided to carry out our G4 analysis in the coding regions of the *RPB1* gene in different evolutionarily distant organisms belonging to the plant kingdom. Although regulation of gene expression is more complex, levels of active RNA polymerase II are important for the overall level of transcription. Therefore, stable G4(s) in the coding sequence of the large subunit of RNA polymerase II could significantly reduce the level of transcription. 

## 2. Results and Discussion

**Figure 1:**
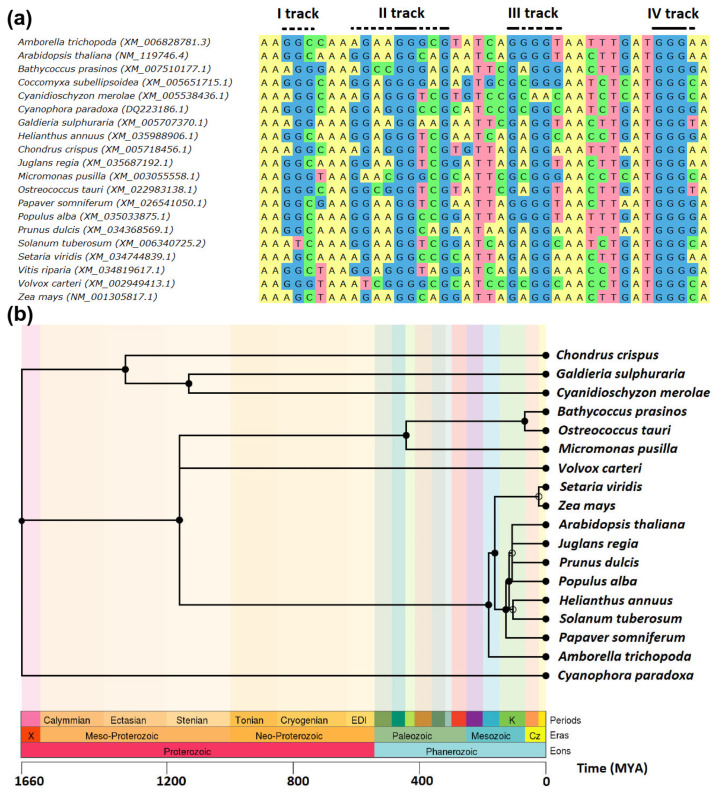
Conserved G4 locus in the *RPB1* gene. (**a**) Multiple sequence alignment of conserved G4-forming loci inside the gene coding for the large subunit of RNA polymerase II (RPB1). The alignment was constructed using the MUSCLE algorithm [34] via UGENE workflow [35]. Mainly the second (II) and fourth (IV) guanine tracks show strong conservation of guanine residues. (**b**) Taxonomic tree with the time of branching estimations (MYA) constructed using the TimeTree tool [36]. *Coccomyxa subellipsoidea* is omitted here because of its unclear phylogeny.

At first, we decided to study the conservation of potential G4-forming RBP1 sequences in evolutionarily distant plants. We aligned the *RPB1* coding sequences of various representative green plants (Viridiplantae) and their closest relative groups, Rhodophyta and Glaucophyta, which all belong to the Archaeplastida supergroup [33]; see Table 1. The alignment revealed a single highly conserved G4 site (Figure 1a), which we inspected further using in silico G4 predictions. It was found that at least 18 of the 20 sequences analyzed had a G4-forming potential.

The identified region was approximately 40 nucleotides long and contained four well-conserved guanine tracks. Their G4 forming potential was verified by four different methods, including QGRS mapper [37]; the G4Hunter [38] algorithm; and the G4RNAscreener web server [39], which comprises cGcC [40] and neural network approaches [41]. Considering the *RPB1* CDS sequence from *Arabidopsis thaliana* (NM_119746.4), the G4 locus started at nucleotide position 1257 and ended at nucleotide position 1296. For the whole genome, the identified region occupies the coordinates of chromosome 4, 16,966,308–16,966,347. The fourth guanine track is 100% conserved, while the other tracks contain a certain plasticity, which can play an important role in the resulting conformation of the formed G4 (Figure 1a). From an evolutionary perspective, it is remarkable that this G4 locus has remained preserved for more than one billion years (Figure 1b). This finding was highly unexpected, because the vast majority of G4 loci are highly divergent and non-conserved, even between closely related species [42,43].

It can be hypothesized that the coding region of the *RPB1* CDS is a priori conserved to maintain the unaltered amino acid sequence of the RNA polymerase II large subunit. Therefore, we inspected the whole *RPB1* CDS (app. 6000 bp) and found that the 40-bp-long potential G4 locus is the most conserved (based on a multiple sequence alignment of *RPB1* gene in plants; details are enclosed in the Supplementary Material S2). Although the G4 locus of the *RPB1* gene is perfectly conserved among evolutionarily distant plant species, its paralogs in *Arabidopsis thaliana* (*rpa1*, *rpc1*, *rpd1a*, and *rpd1b*) have this locus modified by deletions and/or substitutions that disrupt G4-forming potential (see Supplementary Material S3). The largest and catalytic component of RNA polymerase II (RPB1) synthesizes mRNA precursors and many functional non-coding RNAs. RPB1 forms the polymerase active center [44]. Therefore, it is possible that the G4 characterized in our study plays an important regulatory role in vivo by affecting transcription of the *RPB1* gene, thus forming a negative feedback loop, because it is generally accepted that G4s inhibit transcription rates [45,46,47]. Currently, there is also a whole-genome experimental map of G4s in multiple species, including *Arabidopsis thaliana* [48], but no signal for the whole *RPB1* gene was identified via this analysis. We suggest that this could possibly stem from the low-sequencing coverage of this particular site. In addition, only 2407 G4s were mapped across the five *Arabidopsis thaliana* chromosomes using this approach. More specifically, the total number of putative G4 sites in *Arabidopsis thaliana* is supposed to be at minimum five times higher if the strict threshold (1.4) of the G4Hunter prediction algorithm [49] is used. When using data from the *Arabidopsis thaliana* isoform sequencing [50], we found that there is probably an *RPB1* gene isoform comprising exons 1–8 (*RPB1* has 13 exons in total). It is known that G4s can induce premature transcription termination [51]. However, the identified G4 locus is located within exon 6, which is relatively far away from the transcription termination site, so its possible role in this particular event is rather speculative. Nonetheless, various minor non-canonical splice site combinations were recently detected [52].

**Figure 2:**
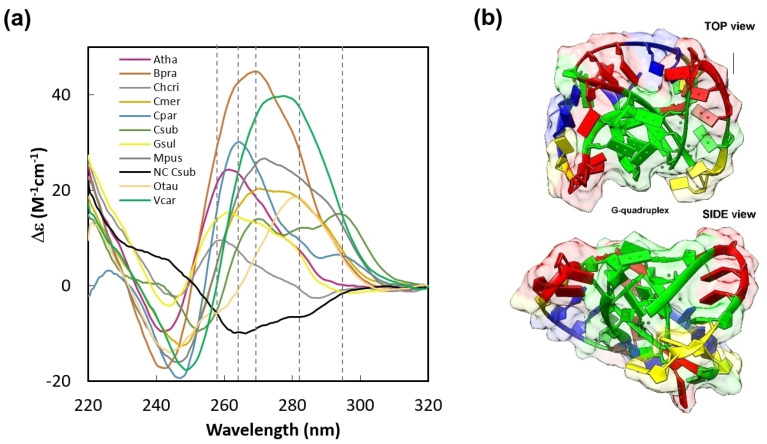
Conformational characterization of conserved G4 locus in the *RPB1* gene. (**a**) The differential CD spectra of the putative G4s in the *RPB1* gene were calculated as the difference of the CD spectra measured at 20 °C and 90 °C. It can be seen that the negative control is different from all other samples analyzed. It also shows different G4 conformations (4–5 CD bands at approx. 258, 264, 270, 282, and 295 nm—grey vertical dashed lines). Atha—*Arabidopsis thaliana*; Bpra—*Bathycoccus prasinos*; Chcri—*Chondrus crispus*; Cmer—*Cyanidioschyzon merolae*; Cpar—*Cyanophora paradoxa*; Csub—*Coccomyxa subellipsoidea*; Gsul—*Galdieria sulphuraria*; Mpus—*Micromonas pusilla*; Otau—*Ostreococcus tauri*; Vcar—*Volvox carteri*; NC—negative control. (**b**) Structural modeling of parallel G4 in *Bathycoccus prasinos*. The coloring of the nucleotides is default NDB (green for guanines, red for adenines, yellow for cytosines, and blue for thymines). The resulting structure in PDB format can be found in Supplementary Material S4.

Next, we inspected the G4-forming potential of selected sequences via circular dichroism (CD) measurements (Figure 2a). All inspected homologous sequences showed clear G4 signatures in differential CD spectra with the characteristic positive peaks at specific wavelength ranges depicted (Figure 2a) by grey vertical dashed lines, whereas the negative control had no significant positive differential CD signal in this spectral region. Interestingly, we found sequence-dependent differences in molar ellipticity across tested species. Such variability might be caused by the different composition of tested sequences, resulting in different folding motifs and, thus, structure. When compared with G4-forming potential (Table 1), only a single discrepancy was identified in the *Cyanidioschyzon merolae* sequence. More specifically, the *RPB1* locus of this unicellular organism has low theoretical potential to form a G4 structure, and, therefore, the obtained CD signal was unexpected. This may be due to the involvement of other nucleotide residues in G4 tetrads (so-called mixed tetrads) that comprise, for example, cytosine residue(s) [53]. Unfortunately, no tool is currently available to determine the G4s formed by other nucleotides than guanine residues. However, existence of such G4s in vitro has been documented [54,55].

To better visualize the structure of a parallel G4 in the *RPB1* gene, we selected one representative sequence from *Bathycoccus prasinos* and modeled its parallel G4 structure in silico. The model is based on information obtained by CD measurement, and it mimics parameters of existing PDB structures using the 3DNus algorithm [56] (Figure 2b).

**Figure 3:**
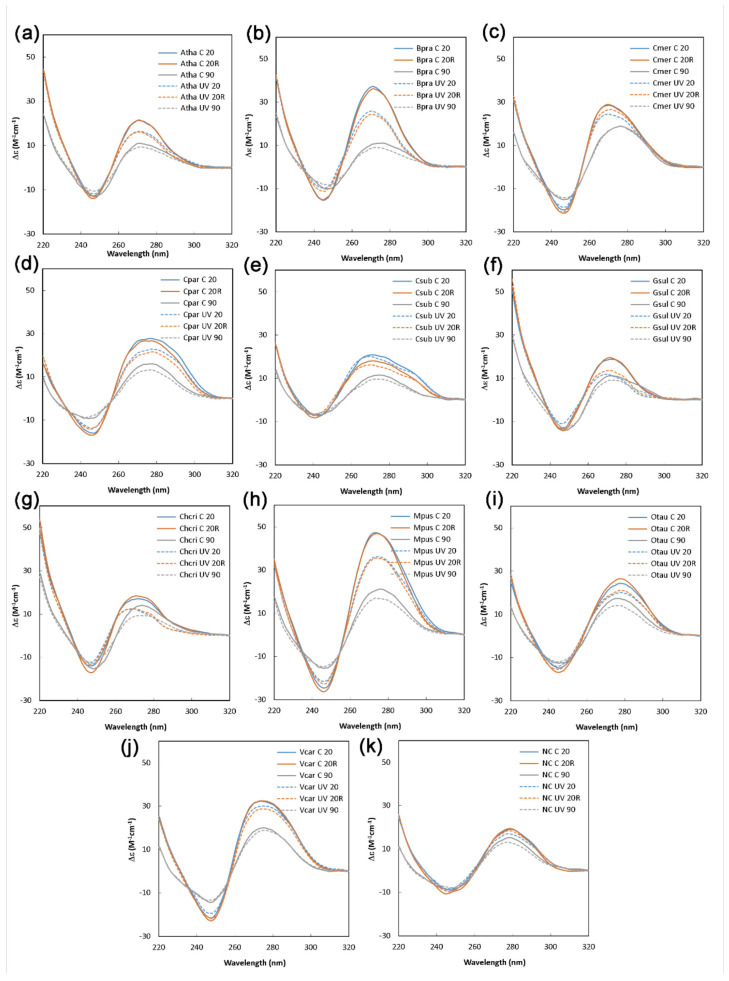
CD spectra of putative G4s in the *RPB1* gene in selected plant species. (**a**) Atha—*Arabidopsis thaliana*; (**b**) Bpra—*Bathycoccus prasinos*; (**c**) Cmer—*Cyanidioschyzon merolae*; (**d**) Cpar—*Cyanophora paradoxa*; (**e**) Csub—*Coccomyxa subellipsoidea*; (**f**) Gsul—*Galdieria sulphuraria*; (**g**) Chcri—*Chondrus crispus*; (**h**) Mpus—*Micromonas pusilla*; (**i**) Otau—*Ostreococcus tauri*; (**j**) Vcar—*Volvox carteri*, (**k**) including negative control (NC). CD spectra were measured at 20 °C (20; blue line), after denaturation (90; grey line) and renaturation (20R; red line) without (C; solid line) or with previous UV irradiations (UV; dashed line). The total exposure was for one hour at 4.1 W/m^2^ of UV-A and 4.1 W/m^2^ of UV-B.

To further validate temperature stability and the reversibility of G4 folding, we performed thermal denaturation followed by the subsequent renaturation and a CD measurement at all three points (Figure 3a–j). Temperatures above 80 °C are generally considered to be enough to melt all common G4 structures [57], and our plots clearly show a decreasing G4 signature in the CD spectrum at 90 °C. After cooling and a short incubation period at 20 °C, the G4 structures renatured, serving as direct evidence of G4 formation. This phenomenon was not observed in the NC sample (Figure 3k).

**Figure 4:**
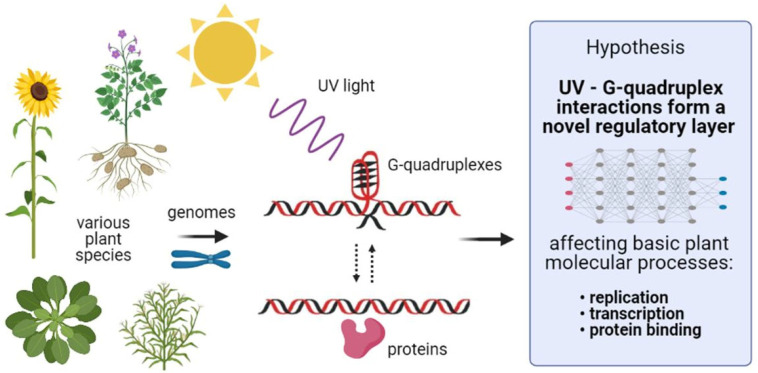
Schematic of UV interacting with G4s. Solar UV radiation penetrates the cell wall, cytoplasmic membrane, and nuclear membrane, and it can directly interact with genomic DNA. We hypothesize that G4s are exceptionally sensitive to UV due to their central metallic K^+^ stabilizing ions and Hoogsteen base pairs forming stacked G4 tetrads. Generally, we propose that the interaction of G4s with UV leads to partial destabilization of the G4 structure and, thus, allows relatively rapid and finely tuned changes of molecular process rates, which affects signaling pathways and plant responses to UV irradiation.

In natural conditions, plants are often exposed to stress factors that may cause substantial DNA damage, such as high soil salinity, drought, or high irradiation. Plants need light for their growth; however, UV light of all wavelengths (UVC, UVB, and even UVA) induces DNA damage, mainly in the form of cyclobutane pyrimidine dimers [58]. Recently, it was found that low-energy UV radiation (266 nm) can photo-ionize human telomeric G-quadruplexes (GGG(TTAGGG)3) in the presence of K^+^ ions in vitro [59]. Here, for the first time, we propose a hypothesis that G4s might function as additional UV sensors, allowing plants to rapidly regulate the rate of DNA replication, gene expression, and protein binding (Figure 4).

**Figure 5:**
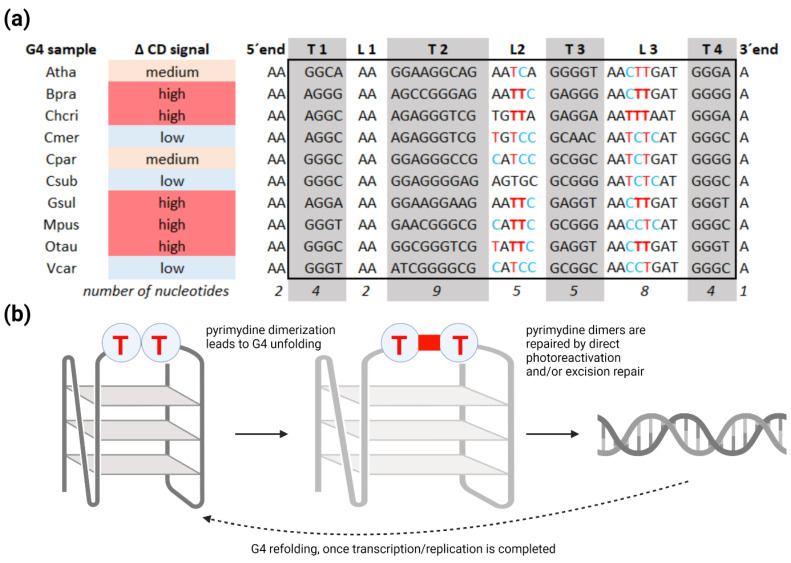
Plants G4s interacts with UVB/UVA light. (**a**) Decrease in molar ellipticity in UV-irradiated G4 samples expressed on the categorical scale (low, moderate, and high decrease). Guanine tracks are highlighted in grey color and designated T1–T4. Loop regions are designated L1–L3. Pyrimidines with the ability to form cyclobutane pyrimidine dimers are depicted in red (thymines) and blue (cytosines). Adjacent thymines with the highest probability to form thymine dimers are in bold. (**b**) Adjacent or opposite pyrimidines in the G4 loops can form cyclobutane pyrimidine dimers [22], which lead to conformational change and/or unfolding of G4 structure. Pyrimidine dimers are then repaired by photoreactivation and/or excision repair [60,61,62], and G4s can then reform via refolding. Concurrently, important molecular processes (DNA replication and transcription) can take place.

To explore our hypothesis of G4 structures being a regulatory element of gene expression in plant cells, we exposed induced G4s to UV for one hour. Interestingly, we found that UV irradiation has a partial inhibitory effect on G4 folding, which is depicted in Figure 3a–j by the dashed lines. It is noteworthy that the decrease in molar ellipticity caused by UV varies between G4-forming oligonucleotides from different plant species. For example, *Cyanidioschyzon merolae* showed a mild decrease, and *Arabidopsis thaliana* showed a medium decrease. In contrast, *Bathycoccus prasinos* or *Micromonas pusilla* displayed a highly pronounced decrease in molar ellipticity associated with G4 presence (Figure 5a). The described variability between plant species is obviously caused by a different nucleotide composition, and, thus, different folding substructures lead to variable G4 sensitivity to UV light. We also confirmed that there were no strand breaks in the oligonucleotides by polyacrylamide gel electrophoresis (PAGE) and that G4s were preserved before and after UV treatment, as verified by thioflavin T (ThT) staining (Supplementary Material S5), which is in accordance with the CD spectroscopy measurements. Figure 5b schematically depicts G4 with adjacent thymines in the loop resulting in thymine dimer formation and G4 structure loosening. Cyclobutane pyrimidine dimers can later be repaired by direct photoreactivation and/or excision repair [60,61,62].

In vivo evidence of G4s has been studied in connection with cancer [63]; genomic instability [64,65]; telomere formation [19]; and the general ability to regulate transcription [66,67], translation [68], and replication [69,70]. It has been shown that chromatin remodeling, which affects G4 formation, can lead to parental loss of chromatin marks [71], showing the important role of epigenetic modifications. Recently, a single-molecule fluorescent probe, which allows visualization of formed G4s in single DNA molecules in living cells, has been developed [72]. Unfortunately, none of these in vivo experiments were, to the best of our knowledge, performed in plants. However, as there is evidence of in vivo G4 formation in different model organisms, we expect that even in in vivo chromatin G4s can form in plants. It has been well documented for several decades that UV-A can induce thymine dimer formation in vivo even in algae [73]; thus, G4s could serve as a sensor for UV radiation. Therefore, their partial disruption could lead to the initiation of specific processes, possibly resulting in the modulation of gene expression. 

## 3. Materials and Methods

### 3.1. Bioinformatics and Structural Modeling

*RPB1* coding regions (CDSs) from 40 model plant species that are evolutionarily distant from one another (Supplementary Material S6) were chosen for the bioinformatic analysis. The MUSCLE algorithm [34] running via UGENE workflow [35] was employed to construct multiple alignments of *RPB1* coding regions (Supplementary Material S7). Analyzed *RPB1* paralogs (FASTA sequences) in *Arabidopsis thaliana* are enclosed in Supplementary Material S8.

The potential to form G4s was predicted via the QGRS mapper [37] and G4screener web server [39], and the resulting scores for the inspected putative G4 sites (obtained by four independent approaches) are enclosed in the supporting data for this article (Supplementary Material S1). The taxonomic tree with the time of branching estimations was constructed using the TimeTree tool [36]. G4 from *Bathycoccus prasinos* was modeled in a 3DNus environment [56] using a supervised approach based on a typical parallel conformation measured by CD assessment. The resulting structure was visualized using UCSF Chimera [74].

### 3.2. Circular Dichroism Measurement

All G4-forming oligonucleotides were purchased in HPLC purity from Elisabeth Pharmacon (Czech Republic) and inducted as reported earlier [75]. CD spectra were recorded in the range of 200–350 nm with a J-815 spectropolarimeter (Jasco, Tokyo, Japan). Spectra were recorded in steps of 0.5 nm with an integration time of 1 s, a bandwidth of 2 nm, and a scanning speed of 50 nm·min^−1^ with 3 accumulations. For all CD analyses, a final concentration of 50 mM KCl was used. To denature the G4 structures, a heating rate of 10 °C·min^−1^ was maintained using a programmable Peltier thermostat up to 90 °C followed by cooling to 20 °C for the CD spectra measurement of renatured G4 structures. A quartz glass cell with a 10 mm path length was used for all CD measurements. The sequence of negative control (NC) was as follows: AAGGGCAAGGAGTGGAGAGTGCGCGTGAATCTCATGTGCAA (designed using the G4Killer tool) [76]. To determine whether the prepared G4 structures have the potential to be a regulatory element, the oligonucleotides were illuminated by a lamp (Philips, TL 20W/12RS UV-B medical, Made in Holland) in a quartz glass cuvette for one hour at 4.1 W/m^2^ UV-A and 4.1 W/m^2^ UV-B radiation. The control and UV-irradiated samples were compared with respect to height of the CD peak (decrease in molar ellipticity for approximately half was judged as high). The decrease in molar ellipticity was computed, and, for later purposes, it was expressed on the categorical scale (low, moderate, and high decrease in molar ellipticity) using the highest and lowest decreases as borders and then evenly divided into these categories. For the detailed spectrum of the UV lamp used in this study, see the Supplementary Material S9. Differential CD spectra are enclosed in Supplementary Material S10. 

### 3.3. Gel Electrophoresis and Thioflavin T Staining

Gel electrophoresis of the selected G4 samples was performed on a nondenaturing 15% acrylamide gel supplemented with 10 mM KCl. The gel was electrophoresed at room temperature (20 °C). After electrophoresis, the gel was stained in a bath of 0.5 µM ThT (which is a widely used fluorescent light-up probe for G4 formation [77]) for 15 min under agitation and then destained for 15 min in an electrophoresis buffer. Gel images were taken on the BioRad ChemiDoc system (Bio-Rad, Hercules, CA, USA) with an automatically optimized exposure time (Supplementary Material S5).

## Figures and Tables

**Table 1:** The most important species inspected in this study (for more species, see Supplementary Material S1). Columns contain their Latin and common names (if applicable, higher and lower taxonomy units, NCBI accession numbers of *RPB1* coding regions, and the presence of predicted conserved G4 sites).

Latin Name/Common Name	Higher Taxonomy Unit	Lower Taxonomy Unit	*RPB1* CDS NCBI ID	Predicted Conserved G4 Site ^1^
*Amborella trichopoda*/ amborella	Viridiplantae	Amborella	XM_006828781.3	Yes
*Arabidopsis thaliana*/ thale cress	Viridiplantae	Brassicales	NM_119746.4	Yes
*Bathycoccus prasinos*	Viridiplantae	Chlorophyta	XM_007510177.1	Yes
*Chondrus crispus*	Rhodophyta	Gigartinales	XM_005718456.1	Yes
*Cyanidioschyzon merolae*	Rhodophyta	Cyanidiales	XM_005538436.1	No
*Cyanophora paradoxa*	Glaucophyta	Cyanophoraceae	DQ223186.1	Yes
*Coccomyxa subellipsoidea*	Viridiplantae	Chlorophyta	XM_005651715.1	Yes
*Galdieria sulphuraria*	Rhodophyta	Cyanidiales	XM_005707370.1	Yes
*Helianthus annuus*/ common sunflower	Viridiplantae	Asterales	XM_035988906.1	Yes
*Juglans regia*/ English walnut	Viridiplantae	Fagales	XM_035687192.1	Yes
*Micromonas pusilla*	Viridiplantae	Chlorophyta	XM_003055558.1	Yes
*Ostreococcus tauri*	Viridiplantae	Chlorophyta	XM_022983138.1	Yes
*Papaver somniferum*/ opium poppy	Viridiplantae	Ranunculales	XM_026541050.1	Yes
*Populus alba*/ white poplar	Viridiplantae	Malpighiales	XM_035033875.1	Yes
*Prunus dulcis*/ almond	Viridiplantae	Rosales	XM_034368569.1	Yes
*Setaria viridis*/ bristlegrass	Viridiplantae	Poales	XM_034744839.1	No
*Solanum tuberosum*/ potato	Viridiplantae	Solanales	XM_006340725.2	Yes
*Vitis riparia*/ riverbank grape	Viridiplantae	Vitales	XM_034819617.1	Yes
*Volvox carteri*	Viridiplantae	Chlorophyta	XM_002949413.1	Yes
*Zea mays*/ maize	Viridiplantae	Poales	NM_001305817.1	Yes

^1^ G4 sites were predicted using four independent approaches. For more details, see the Supplementary Material S1.

## Data Availability

All supporting data generated for this manuscript are freely available here together with a short commentary: https://zenodo.org/record/4541573#.YCrckWj0lPY (accessed on 7 July 2021).

## Supplementary Materials

The following are available online at https://zenodo.org/record/4973514#.YMtNZqj7RPY (accessed on 7 July 2021). Supplementary Material S1: Analyzed *RPB1* sequences in 40 plant species together with detailed characteristics and G4 prediction using four different computational approaches. Supplementary Material S2: G4 locus is the most conserved within the *RPB1* gene. Supplementary Material S3: Multiple alignment of G4 locus in *RPB1* paralogs (*Arabidopsis thaliana*). Supplementary Material S4: Modelled 3D structure of G4 from *Bathycoccus prasinos*. Supplementary Material S5: Gel electrophoresis and ThT staining of the selected G4-forming sequences. Supplementary Material S6: All analyzed *RPB1* sequences in FASTA format. Supplementary Material S7: Aligned *RPB1* sequences in FASTA format. Supplementary Material S8: *RPB1* paralogs in *Arabidopsis thaliana*. Supplementary Material S9. Spectral composition of light used in the UV experiment. Analysis of emitted light was performed using an Ocean Optics (HR4000CG-UV-NIR, USA) device. Supplementary Material S10: Differential CD spectra—comparison without and with previous UV treatment.
